# Precipitation Evolution in the Austenitic Heat-Resistant Steel HR3C upon Creep at 700 °C and 750 °C

**DOI:** 10.3390/ma15134704

**Published:** 2022-07-05

**Authors:** Liming Xu, Yinsheng He, Yeonkwan Kang, Jine-sung Jung, Keesam Shin

**Affiliations:** 1 School of Materials Science and Engineering, Changwon National University, Changwon 51140, Korea; xulimings2016@gmail.com; 2 KEPCO Research Institute, Korea Electric Power Corporation, Daejeon 34056, Korea; yinshenghe@kepco.co.kr (Y.H.); yeonkwan.kang@kepco.co.kr (Y.K.); jinesung.jung@kepco.co.kr (J.-s.J.)

**Keywords:** HR3C steel, microstructure, creep rupture, precipitates

## Abstract

HR3C (25Cr-20Ni-Nb-N) is a key material used in heat exchangers in supercritical power plants. Its creep properties and microstructural evolution has been extensively studied at or below 650 °C. The precipitation evolution in HR3C steel after creep rupture at elevated temperatures of 700 °C and 750 °C with a stress range of 70~180 MPa is characterized in this paper. The threshold strength at 700 °C and 750 °C were determined by extrapolation method to be $\sigma_{10^{5}}^{700}=$ 57.1 MPa and $\sigma_{10^{5}}^{750}=$37.5 MPa, respectively. A corresponding microstructure investigation indicated that the main precipitates precipitated during creep exposure are Z-phase (NbCrN), M_23_C_6_, and σ phase. The dense Z-phase precipitated dispersively in the austenite matrix along dislocation lines, and remained stable (both size and fraction) during long-term creep exposure. M_23_C_6_ preferentially precipitated at grain boundaries, and coarsening was observed in all creep specimens with some continuous precipitation of granular M_23_C_6_ in the matrix. The brittle σ phase formed during a relatively long-term creep, whose size and fraction increased significantly at high temperature. Moreover, the σ phases, grown and connected to form a large “island” at triple junctions of grain boundaries, appear to serve as nucleation sites for high stress concentration and creep cavities, weakening the grain boundary strength and increasing the sensitivity to intergranular fracture.

## 1. Introduction

HR3C (25Cr-20Ni-Nb-N) is an advanced austenitic steel, used as a superheater and reheater in ultra-supercritical (USC) boilers for its high-temperature oxidation resistance and outstanding creep strength [1,2]. Nevertheless, the microstructural degradation, especially precipitation behavior, inevitably affects the mechanical properties during long-term creep or exposure to temperatures of 600 °C and above.

In austenitic heat-resistant steel, a large number of research works reveal that the types of precipitates during long-term service exposure or creep are mainly NbCrN (Z-phase), M_23_C_6_, and σ phase at 600 °C or 650 °C. The precipitation and growth of different precipitates are closely related to the microstructural stability and mechanical properties [3,4,5]. Z-phase is a typical precipitate with a tetragonal structure with a = 0.3073 nm and b = 0.7391 nm, which has a strong hardening effect on the performance of the HR3C steel [6]. The fine dispersion of the Z-phase can pin dislocations and increase the strength, as Golańsk et al. and Bin, W et al. reported [7,8]. M_23_C_6_ carbide, as a metastable phase, is preferentially precipitated at the grain boundaries in the early stage of service exposure [9]. Zhang et al. discovered the growth and coarsening of M_23_C_6_ at the grain boundary, increasing the tendency of intergranular cracking [10]. The FeCr-type σ phase has a tetragonal structure with a = 0.88 nm and b = 0.45 nm, which is a common precipitate in stainless steels such as AISI304, AISI321, AISI347, and other similar types [11,12,13]. The presence of the σ phase greatly decreases the plasticity, toughness strength, and corrosion resistance of heat-resistant steel after long-term service, as Cao et al. reported [14].

However, since the temperature of the superheater and reheater in practical applications can be overheated to above 650 °C, additional research on the relationship between microstructural evolution and mechanical properties at a more elevated temperature is essential. In this study, microscopic observation and phase analysis were used to investigate the effect of various precipitates on the creep behaviors at temperatures up to 700 °C and 750 °C under different stresses.

## 2. Materials and Methods

In this work, the as-received HR3C boiler tube steel was domestically manufactured with the following specifications: outer diameter of 57 mm and wall thickness of 4.5 mm. The chemical composition of HR3C steels in this study is listed in Table 1.

The samples for creep testing were manufactured with a diameter of 4 mm and gauge length of 25 mm according to ASTM E8. In accordance with ASTM E 139-11, the creep tests were carried out at 700 °C and 750 °C under different stresses of 70~180 MPa with the creep tester (ATS arm ratio creep tester, Series 2320 Lever Arm). The temperature of the samples was monitored by thermocouple and controlled by an induction heating system.

The microstructure was analyzed by JSM-6510 scanning electron microscope (SEM) (JEOL Ltd., Tokyo, Japan, equipped with INCA EDS, operated at an accelerating voltage of 20 kV), JSM-7900F high-resolution scanning electron microscope (HRSEM) (JEOL Ltd., Tokyo, Japan, equipped with EDS), and Philips CM200 transmission electron microscope (TEM) (FEI Ltd., Hillsboro, OR, USA, operated at 200 kV). Specimens for SEM were prepared by mechanical grinding, polishing, and final etching with Kalling’s #2 reagent. Specimens for ECCI (Electron Channeling Contrast Imaging) and TEM observation were prepared by mechanical grinding, polishing, and final electrolytic twin-jet polishing (Struers TenuPol-5) with a solution of 10% perchloric acid in ethanol at 15 V for 40 s. The microhardness was tested by a Vickers hardness tester (Future-Tech. JP/FM-7) under a load of 200 gf for more than 50 iterations for each specimen.

## 3. Results and Discussion

### 3.1. Creep Rupture Test

The specific conditions and results of the creep tests are displayed in Table 2.

**Figure 1:**
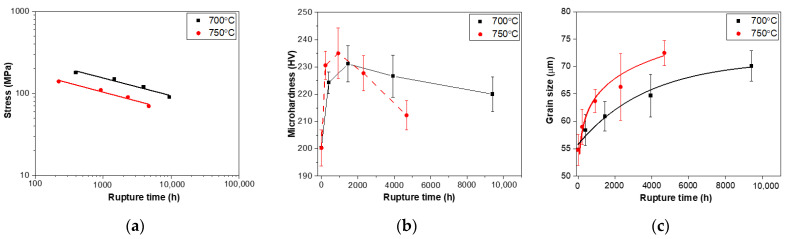
(**a**) The log-log plot of stress versus rupture time, (**b**) microhardness versus rupture time, and (**c**) grain size versus rupture time.

The relationship between the applied stress and microhardness as a function of time till rupture is shown in Figure 1. The curve in Figure 1a shows that the long-term creep rupture strengths of HR3C can be expressed by linear regression:

σ = A log t + B(1)

where t is the creep rupture time, σ is the applied stress, and A and B are constants which are related to the material and test temperature. Therefore, the threshold strength of HR3C steel at 700 °C and 750 °C after 10^5^ h creep test can be determined by the extrapolation method as follows: $\sigma_{10^{5\;}}^{700}$= 57.1 MPa, $\sigma_{10^{5}}^{750}\;$= 37.5 MPa. According to the safety requirement of ASTM standard (KA-SUS310J1 TB) [15], the threshold strength for 10^5^ h is 55 MPa at 700 °C and 25 MPa at 750 °C.

Figure 1b shows the variation of Vickers microhardness with rupture time. The microhardness increases rapidly at the early stages of the creep test and reaches the maximum value of 231 HV at 700 °C/1463 h and 235 HV at 750 °C/2310 h. Then, the microhardness decreases again with prolonged creep duration. The early increase in microhardness results from the continuous precipitation of M_23_C_6_ in the early stage of the creep.

The average grain size of HR3C steel was measured with low-magnification optical microscope images by the average grain intercept (AGI) method according to ASTM E112-13. The variation in grain size is shown in Figure 1c below. According to the ideal grain growth,

$$d^{2}-d_{0}{}^{2}=\mathrm{kt},$$

where *d*_0_ is the initial grain size, *d* is the final grain size and k is a temperature-dependent constant given by an exponential law:

(2) $$k=k_{0}\exp\;(-Q/\mathrm{RT}),$$

where *k*_0_ is a constant, T is the absolute temperature and Q is the activation energy for boundary mobility.

The calculated value of k with the fitting curves is 0.162 and 0.372 at 700 °C and 750 °C, respectively, which means the grain growth rate at 750 °C is much faster than at 700 °C.

#### 3.1.1. Fracture Morphology

**Figure 2:**
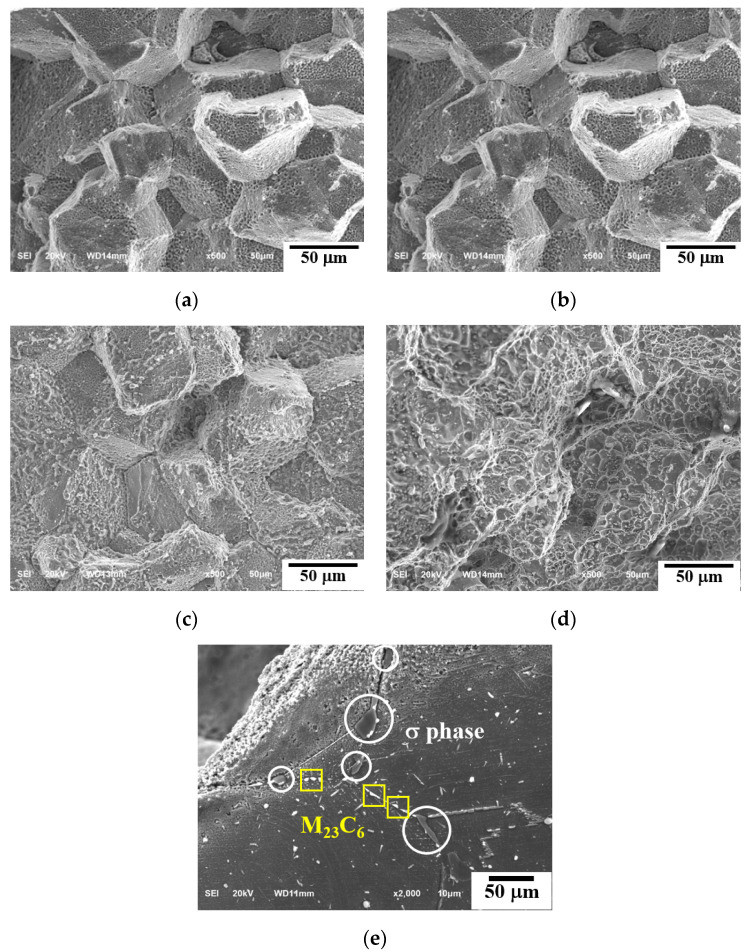
SEM micrographs of crept fractures: (**a**) 700 °C/180 MPa/398 h, (**b**) 700 °C/150 MPa/1463 h, (**c**) 700 °C/120 MPa/3945 h, (**d**) 700 °C/90 MPa/9411 h, and (**e**) electropolished fracture surface of (**d**).

**Figure 3:**
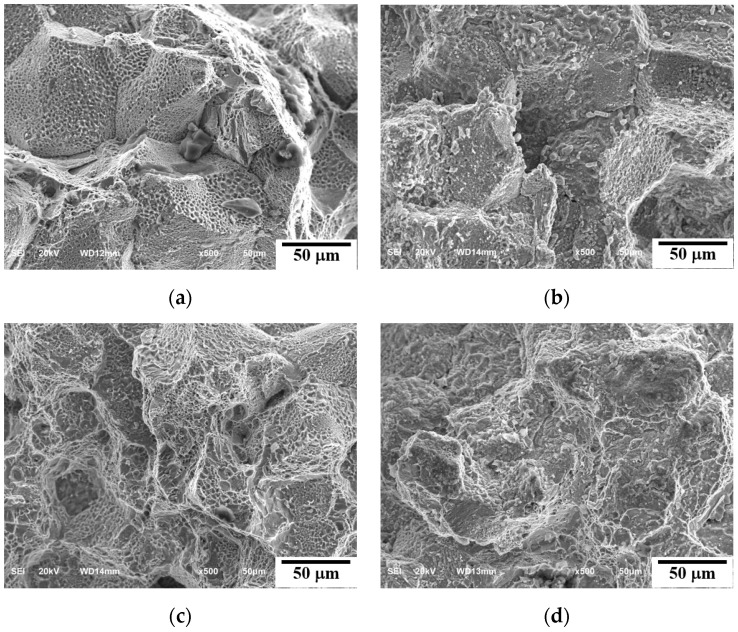
SEM micrographs of crept fractures: (**a**) 750 °C/150 MPa/222 h, (**b**) 750 °C/110 MPa/925 h, (**c**) 750 °C/90 MPa/2310 h, and (**d**) 750 °C/70 MPa/4680 h.

SEM micrographs of the rupture surface at 700 °C and 750 °C are shown in Figure 2 and Figure 3, respectively. Figure 2a–c and Figure 3a,b show a totally intergranular brittle fracture feature with cleavage and a rock candy fracture surface. However, with decreasing applied stress and increasing time to rupture, a dimpled morphology appears on the grain facets at 700 °C/90 MPa/9411 h in Figure 2d and 750 °C/90 MPa/2310 h, 750 °C/70 MPa/4680 h in Figure 3c,d. Creep is a time-dependent deformation under a constant load or stress at elevated temperatures. Generally, the creep of a metal has three stages. When a high stress is applied, there is no steady stage (secondary creep stage) of continuous microstructural changes under the service condition. The growth of M_23_C_6_ and σ phase at the grain boundaries and the continuous precipitation of granular M_23_C_6_ in the matrix occur at a low creep rate in the second creep stage. Coarse M_23_C_6_ and σ phase at the grain boundaries serve as high stress concentration and creep cavity nucleation sites, leading to the propagation of cracks on the grain boundaries and, eventually, intergranular fracture. Some partial dimple morphology formed on the fracture facets due to the cavity developed on the surface of M_23_C_6_ surrounded by the ductile matrix [16]. Figure 2e, the electropolished fracture surface, shows large fractured σ phases on the grain boundary, indicating the σ phases are insignificant to the strengthening.

#### 3.1.2. Precipitation Behavior

**Figure 4:**
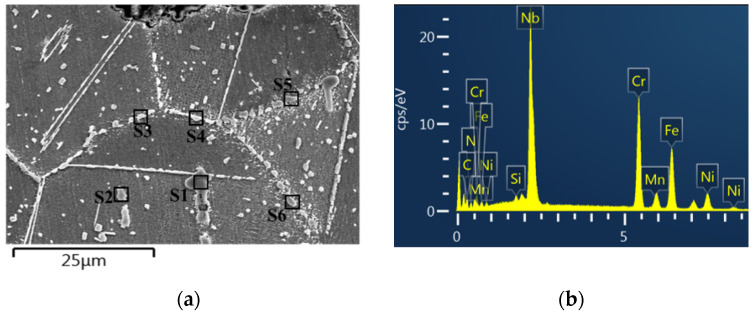

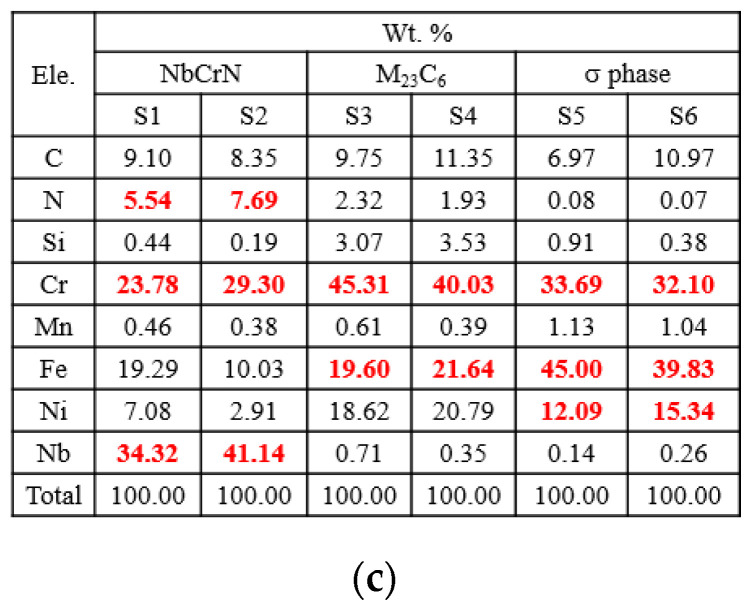
EDS analysis of precipitates in the 700 °C/90 MPa/9411 h crept specimen: (**a**) SEM micrographs in the near-fracture region, (**b**) the corresponding spectrum of site 1 (S1) and (**c**) EDS results of S1~S6 in (**a**) with red letters for major elements.

Typical precipitates of HR3C steel after the creep rupture test and the corresponding EDS results are shown in Figure 4. The EDS shows excessive precipitation at the grain boundaries and inside the grains. The large undissolved particles inside the grains are Cr- and Nb-rich nitrides at site 1 and site 2 (S1 and S2) in Figure 4a, and are the primary Z-phase. The continuous chain-like precipitates in the grain boundary are identified as Cr-rich M_23_C_6_ carbides at site 3 and site 4 (S3 and S4) in Figure 4a. In addition, the large blocky particles (~2 μm) are Fe- and Cr-rich σ phases at site 5 and site 6 (S5 and S6).

**Figure 5:**
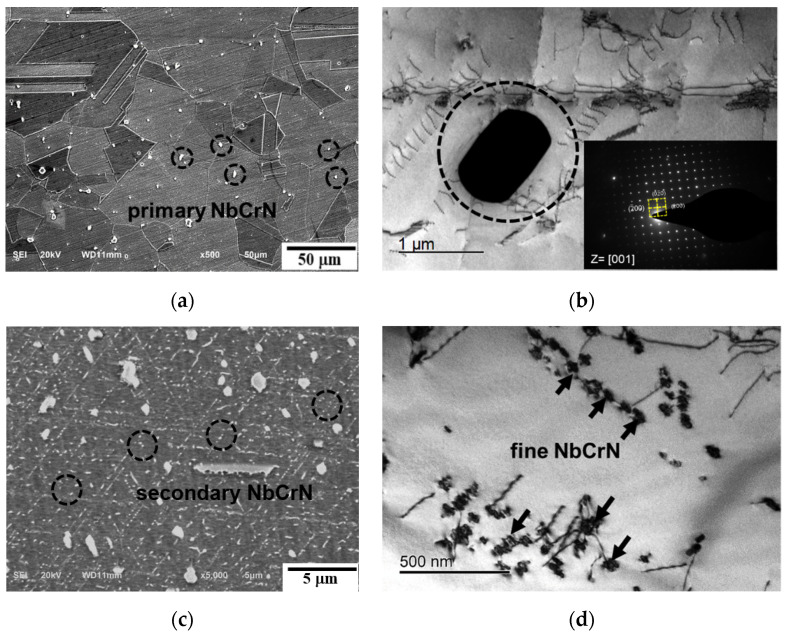
SEM and TEM micrographs of Z-phase in the specimen of: (**a**,**b**) as-received, (**c**,**d**) 700 °C/90 MPa/9411 h.

Figure 5 shows the Z-phase particles in the as-received and creep rupture specimens of 700 °C/90 MPa/9411 h. In the as-received specimen, the Z-phase precipitated in the austenite matrix uniformly, as shown in Figure 5. These coarse undissolved particles are considered as primary Z-phase with a size of ~1 μm. This was also reported by Zieliński, A [17]. In specimens with longer times to rupture, a fine Z-phase is observed, as shown in Figure 5c,d, also called the secondary Z-phase, with a size of ~50 nm. By interacting with the dislocations, present in high density, this fine dispersion of Z-phase enhances the strength of the matrix, as reported by Hu et al. [18].

**Figure 6:**
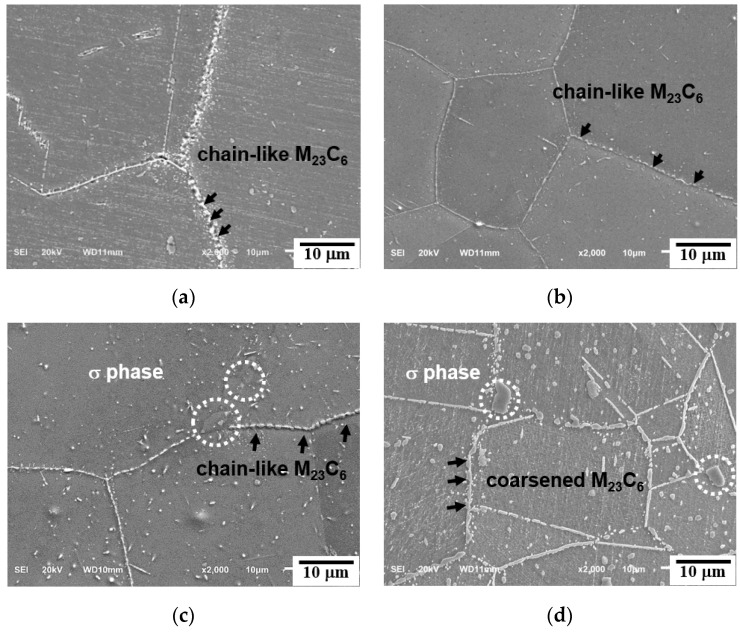
SEM micrographs from cross-sectional fractures of creep specimens: (**a**) 700 °C/180 MPa/398 h, (**b**) 700 °C/150 MPa/1463 h, (**c**) 700 °C/120 MPa/3945 h, and (**d**) 700 °C/90 MPa/9411 h.

**Figure 7:**
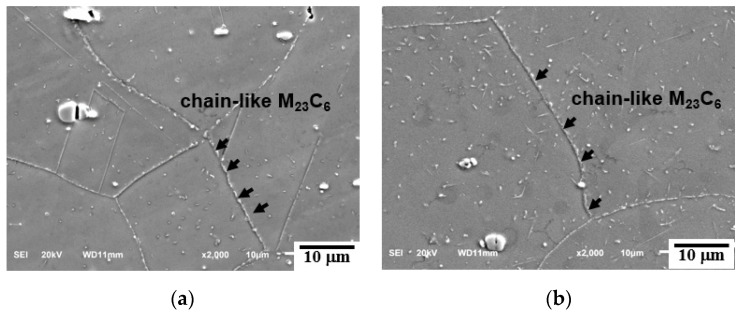

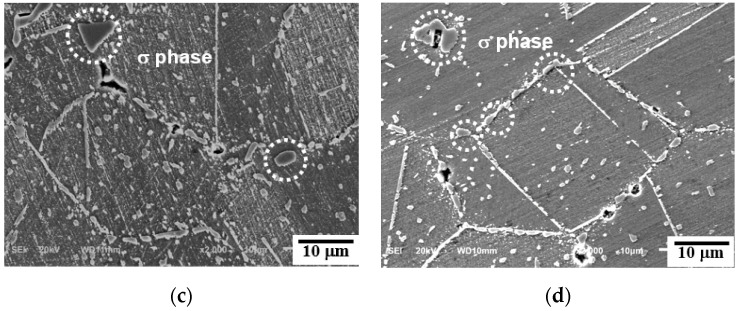
SEM micrographs from cross-sectional fractures of creep specimens: (**a**) 750 °C/150 MPa/222 h, (**b**) 750 °C/110 MPa/925 h, (**c**) 750 °C/90 MPa/2310 h, and (**d**) 750 °C/70 MPa/4680 h.

The SEM micrographs of a triple junction of the grain boundaries of creep-ruptured specimens at 700 °C and 750 °C are shown in Figure 6 and Figure 7. The M_23_C_6_ precipitation is a diffusion-type phase transformation controlled by the driving force for nucleation and the diffusion of the C and Cr atoms in the austenite steel. Therefore, as shown in Figure 6a and Figure 7a, the rod-like M_23_C_6_ particles (~200 nm) preferentially precipitated at the grain boundaries at the early stage of creep due to the higher interfacial energy of grain boundaries and its higher atom diffusion rate compared to those of the grain interiors [19,20]. With prolonged creep time, the M_23_C_6_ particles at the grain boundaries coarsened (up to ~600 nm) and gradually grew into chains, thereby decreasing the pinning efficiency. Meanwhile, the granular M_23_C_6_ particles (~200 nm) continuously precipitated in the matrix with the extension of the creep rupture time.

**Figure 8:**
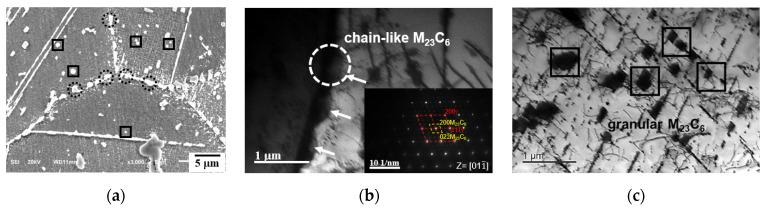
Distribution of M_23_C_6_ in the 9411 h crept specimen: (**a**) SEM micrographs of two kinds of M_23_C_6_, (**b**) TEM micrographs of coarsened M_23_C_6_ distributed along the grain boundary, and (**c**) TEM micrographs of fine M_23_C_6_ in the grain interior. Precipitates in dotted circles indicate chain-like M_23_C_6_ at the grain boundary and precipitates in solid rectangles indicate granular M_23_C_6_ in the grain interior.

Figure 8 shows the distribution of M_23_C_6_ in the 9411 h crept specimen. The chain-like M_23_C_6_ carbides distributed along the grain boundaries and fine granular M_23_C_6_ carbides precipitated in the grain interior are shown in Figure 8b and Figure 8c, respectively. Consistent with Figure 6a and Figure 7a, the preferred precipitation of M_23_C_6_ carbides at the grain boundaries occurred due to the grain boundary having a higher interfacial energy and a faster diffusion rate for alloying atoms than in the grain interior [21]. The M_23_C_6_ carbides precipitated in the grain boundaries could provide good creep resistance due to the pinning effects of grain boundaries at the early stage of creep. However, M_23_C_6_ carbide is metastable, with low thermodynamic stability [22]. The coarsening of M_23_C_6_ carbides noticeably weakened the grain boundaries and increased the risk of embrittlement. In addition, the growth of Cr-rich M_23_C_6_ consumed Cr from the matrix and contributed to the formation of a Cr-depleted region near the grain boundaries, resulting in intergranular corrosion [23,24,25]. On the other hand, the fine M_23_C_6_ precipitated in the grain interior increased the strength of the matrix through precipitate hardening and preventing the motion of dislocations according to the Orowan law, as shown in Figure 8c.

**Figure 9:**
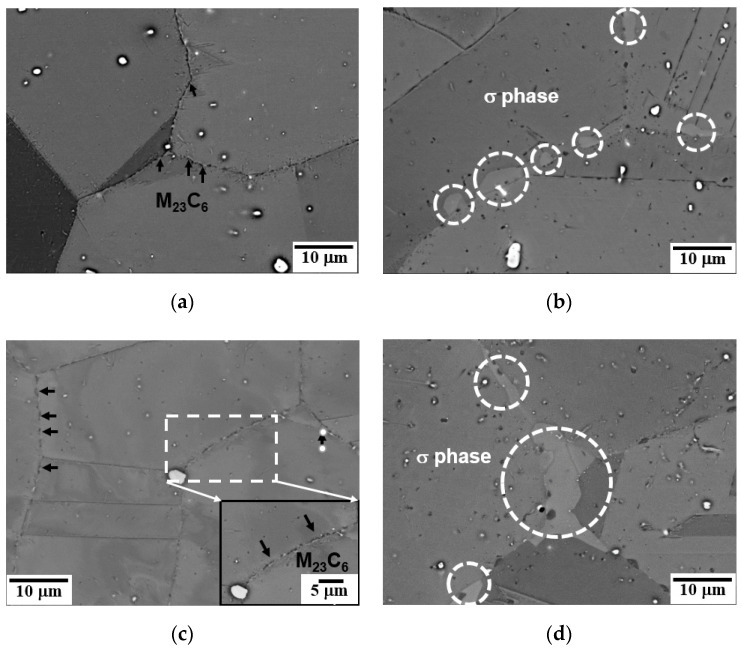
ECCI micrographs from cross-sectional fractures of creep specimens: (**a**) 700 °C/180 MPa/398 h, (**b**) 700 °C/90 MPa/9411 h, (**c**) 750 °C/150 MPa/222 h, and (**d**) 750 °C/70 MPa/4680 h.

The ECCI micrographs of precipitations in the grain boundaries are shown in Figure 9. It can be seen that only M_23_C_6_ carbides are observed in the grain boundaries at the beginning of the creep process, Figure 9a,c. At creep times of 3945 h at 700 °C and 2310 h at 750 °C, the blocky σ phase was observed at the grain boundaries. It can be seen that the blocky σ phase grew and connected to a larger σ phase “island”, serving as nucleation sites for high stress concentration and creep cavities and leading to the propagation of cracks on the grain boundaries and an eventual intergranular fracture, as shown in Figure 9d.

**Figure 10:**
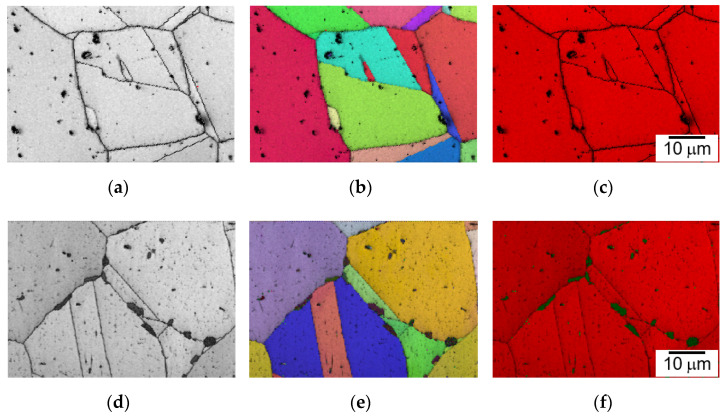

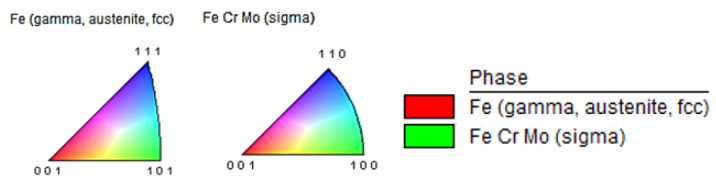
EBSD micrographs of 398 h crept specimen: (**a**) image quality, (**b**) inverse pole figure, and (**c**) phase map with grain boundary; and 9411 h crept specimen with σ phase: (**d**) image quality, (**e**) inverse pole figure, and (**f**) phase map with grain boundary.

The EBSD micrographs of 398 h and 9411 h crept specimens at 700 °C are shown in Figure 10. As shown in Figure 10a–c, there is no σ phase observed at 398 h. At 9411 h, the blocky σ phase formed along the high-angle grain boundaries, as presented in Figure 10d–f.

**Figure 11:**
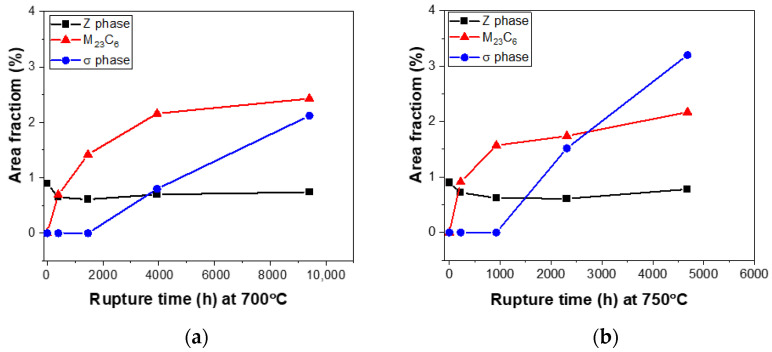
Precipitation evolution of Z-phase, M_23_C_6_, and σ phase at (**a**) 700 °C and (**b**) 750 °C.

Figure 11 shows the area fractions of Z-phase, M_23_C_6_, and σ phase at the two temperatures. Note that the fraction of Z-phase changes little with creep time and temperature compared to the M_23_C_6_ and σ phase due to its slower nucleation and growth rate [10]. In addition, the size of the fine Z-phase remains s at ~50 nm. In contrast, the fraction of M_23_C_6_ and σ phase grow linearly with creep time. The growth rate of M_23_C_6_ gradually decreases at both temperatures. This has two main reasons: (1) though the preferred M_23_C_6_ precipitate at the grain boundaries is easily coarsened with increasing creep time, the fine M_23_C_6_ carbides in the grain interior are stable; (2) the precipitation of σ phase consumes the Fe and Cr, presumably suppressing the formation of M_23_C_6_ carbides [11]. The early increase in microhardness results from the continuous precipitation of M_23_C_6_ in the early stage of the creep as shown in Figure 2b. The growth of the σ phase is linear at the grain boundaries (the first three data points are σ phase free). The growth rate of σ phase at 750 °C is almost twice that at 700 °C.

In summary, the microstructural evolution of HR3C in this study can be characterized as follows: (1) before the creep test, the HR3C specimen had a Z-phase distributed both on the boundary and in the interior of the grains, whereas both the σ phase and M_23_C_6_ were not present; (2) the creep-ruptured specimens had high-density M_23_C_6_ precipitated mostly at the grain boundary from the early stage of the creep test, and grain interior M_23_C_6_ appeared at the later stage of the creep test; σ phases (up to 10 μm) were found mostly at the grain boundary, whereas the Z-phase appeared very stable and did not show much difference compared to the before test. The coarse σ phases were observed as fractured at the creep rupture surface.

## 4. Conclusions

Standard creep rupture strength tests were carried out for HR3C steel at 700 °C and 750 °C. The results show that the HR3C steel in this study has good creep performance. The major precipitates in this alloy, i.e., Z-phase, M_23_C_6_, and σ phase, are densely distributed on and along the grain boundaries, with some presence in the grain interior. The nucleation and growth of these phases have significant effects on the creep rupture behavior:

1. The dense Z-phase, including primary coarse Z-phase (~1 μm) and secondary fine Z-phase (~50 nm), dispersively precipitated in the matrix along the dislocation lines. Moreover, it showed high stability (both the size and the area fraction of ~0.8%) against coarsening with the extension of creep time;
2. The M_23_C_6_ preferentially precipitated at the grain boundaries and coarsened distinctly from ~200 nm to ~600 nm after a creep rupture of 9411 h. Meanwhile, granular M_23_C_6_ continuously precipitated in the matrix with the extension of creep rupture time and kept a relatively stable size of ~200–300 nm under long-term creep exposure;
3. The σ phase did not observe in the early stage of creep exposure till 700 °C/120 MPa/3945 h and 750 °C/120 MPa/2310 h. The fraction of the σ phase grew linearly with increasing time to rupture and the growth rate of σ phase at 750 °C was higher than at 700 °C;
4. All the crept HR3C specimens showed the intergranular brittle fracture under different stresses. As the time to creep rupture increased (low creep stress), partial dimple morphology formed on fracture facets by the void nucleation of M_23_C_6_. Coarse M_23_C_6_ and σ phase at the grain boundaries served as nucleation sites for high stress concentrations and creep cavities and led to the propagation of cracks on the grain boundaries and an eventual intergranular fracture;
5. Creep rupture mechanism and corrosion: the creep rupture specimens showed a typical intergranular fracture with small dimples caused by the decoupling of M_23_C_6_, indicating that the grain boundary was weakened due to the dense precipitation of this phase, even though the matrix was ductile enough to show dimples. The coarse and brittle σ phases do not play any significant role in strengthening. The depletion of Cr in the periphery of the grain boundary is expected to be a cause of corrosion.

## Figures and Tables

**Table 1:** Chemical composition of the HR3C steel (wt. %).

Element	Cr	Ni	Nb	C	N	Mn	Si	Fe
ASTM A213	24.0~ 26.0	19.0~ 22.0	0.20~ 0.60	0.04~ 0.10	0.15~ 0.35	2.00 max	1.50 max	Balance
* EDS result	24.4	18.4	0.4	5.9	0.3	1.2	0.4	49.0

* EDS is carried out with a large area signal acquisition mode.

**Table 2:** Creep test conditions and time till rupture.

State	Stress (MPa)	Time (h)
As-received	-	-
700 °C	180	398
	150	1463
	120	3945
	90	9411
750 °C	140	222
	110	925
	90	2310
	70	4680

## Data Availability

Not applicable.
